# PDI inhibitor LTI6426 enhances panobinostat efficacy in preclinical models of multiple myeloma

**DOI:** 10.1007/s00280-022-04425-3

**Published:** 2022-04-05

**Authors:** Reeder M. Robinson, Ashton P. Basar, Leticia Reyes, Ravyn M. Duncan, Hong Li, Nathan G. Dolloff

**Affiliations:** 1 Department of Cellular and Molecular Pharmacology and Experimental Therapeutics, Medical University of South Carolina, 173 Ashley Ave, MSC509, Charleston, SC 29425 USA; 2 Department of Public Health Sciences, Medical University of South Carolina, Charleston, SC USA; 3 Hollings Cancer Center, Medical University of South Carolina, Charleston, SC USA

**Keywords:** Multiple myeloma, Panobinostat, Protein disulfide isomerase, Epigenetic therapy, Histone deacetylase

## Abstract

The histone deacetylase inhibitor (HDACi), panobinostat (Pano), is approved by the United States Food and Drug Administration (FDA) and European Medicines Agency (EMA) for treatment of relapsed/refractory multiple myeloma (MM). Despite regulatory approvals, Pano is used on a limited basis in MM due largely to an unfavorable toxicity profile. The MM treatment landscape continues to evolve, and for Pano to maintain a place in that paradigm it will be necessary to identify treatment regimens that optimize its effectiveness, particularly those that permit dose reductions to eliminate unwanted toxicity. Here, we propose such a regimen by combining Pano with LTI6426, a first-in-class orally bioavailable protein disulfide isomerase (PDI) inhibitor. We show that LTI6426 dramatically enhances the anti-MM activity of Pano in vitro and in vivo using a proteasome inhibitor resistant mouse model of MM and a low dose of Pano that exhibited no signs of toxicity. We go on to characterize a transcriptional program that is induced by the LTI6426/Pano combination, demonstrating a convergence of the two drugs on endoplasmic reticulum (ER) stress pathway effectors *ATF3* (Activating Transcription Factor 3)*, DDIT3*/CHOP (DNA Damage Inducible Transcript 3, a.k.a. C/EBP Homologous Protein)*,* and *DNAJB1* (DnaJ homolog subfamily B member 1, a.k.a. HSP40)*.* We conclude that LTI6426 may safely enhance low-dose Pano regimens and that *ATF3,* DDIT3/CHOP*,* and *DNAJB1* are candidate pharmacodynamic biomarkers of response to this novel treatment regimen.

## Introduction

Global histone methylation and acetylation patterns are abnormal in MM and cancer as a whole [1–4]. Epigenetic dysfunction has therefore been implicated in MM disease etiology and epigenetic regulators such as HDACs have been proposed as therapeutic targets. Panobinostat (Pano), is a pan-HDACi that was approved by the FDA and EMA for treatment of relapsed/refractory MM in combination with the proteasome inhibitor bortezomib (Btz) and dexamethasone [5–7]. Pano potently inhibits nearly all classes of HDAC enzymes with IC_50_ values in the low nanomolar range and dramatically alters histone acetylation and gene transcription patterns in cells [8]. Despite the strengths of Pano as a drug candidate, its clinical impact has been limited compared to other novel MM agents. This is due in part to grade 3/4 toxicities including hematological adverse events (AEs), diarrhea, and fatigue [7, 9, 10], although these AEs apparently result from the combination with Btz, as they are far less frequent and severe with Pano monotherapy [10–12]. It is possible that Pano combinations with other standard of care MM agents could offer benefits to patients with improved tolerability. For example, recent studies using a steroid-sparing regimen combining Pano with the second generation proteasome inhibitor, carfilzomib [13, 14], demonstrated efficacy with limited toxicity and dosing flexibility. Therefore, it is possible that the optimal clinical context for Pano in the complex MM treatment paradigm is still evolving [15]. Additional work in this area could reveal a more optimal clinical setting for Pano, and it is our position that further studies are warranted considering the large body of work implicating epigenetic dysregulation in MM.

In this study we propose a new therapeutic strategy that could enhance the anti-MM activity of Pano while also permitting dose reduction to mitigate unwanted side effects. Specifically, we demonstrate the preclinical potential of combining Pano with the protein disulfide isomerase (PDI) inhibitor, LTI6426 (formerly E64FC26) [16–19]. LTI6426 is an orally bioavailable pan-isoform inhibitor of PDI that has potent anti-MM activity as a single agent and restores proteasome inhibitor sensitivity in resistant MM cells [16, 17]. PDI possesses two redox active catalytic centers that mediate oxidative protein folding within the endoplasmic reticulum (ER). Specifically, PDI catalyzes the oxidation of cysteine residues in client proteins and introduces and isomerizes inter- and intrachain disulfide bonds to give proteins their proper tertiary and quaternary structures [20, 21]. The dual role of PDI as a regulator of ER and oxidative stress pathways strikes two vulnerabilities of MM plasma cells. Normal and malignant plasma cells are capable of producing thousands of immunoglobulin (Ig) proteins per second [22]. Igs are rich in disulfide linkages and their formation generates equimolar quantities of reactive oxygen species. Similarly, high protein production rates are accompanied by the accumulation of misfolded/unfolded proteins leading to high basal levels of ER stress [23, 24]. Thus, the dual function of PDI makes it an appealing drug target in MM, and we’ve shown that PDI inhibitors are effective when combined with drugs targeting the ubiquitin–proteasome system. In addition to synergy with Btz and other proteasome inhibitors, we found, somewhat unexpectedly, that LTI6426 was also highly synergistic with HDACi but not ~ 150 other marketed oncology drugs in solid tumor lines [18]. In the current study, we explored the potential of an LTI6426/Pano combination in MM given that it is FDA-approved but underutilized in this indication. We show a synergistic interaction between LTI6426 and Pano as well as other pan and isoform selective HDACi, but not other epigenetic therapies. We went on to characterize the molecular events underlying the anti-MM response to LTI6426/Pano, which induces synergistic transcriptional upregulation of key effectors in the ER stress pathway including *ATF3*, *DDIT3*/CHOP, and *DNAJB1*. We extended our findings to test LTI6426 with Pano in vivo using a mouse model of treatment resistant MM in a multidrug cocktail that also includes Btz, which is representative of the FDA-approved regimen for Pano in relapsed/refractory MM. The three drug cocktail used a low dose of Pano that was well tolerated in vivo and LTI6426 significantly enhanced the Pano regimen to improve animal survival. We validated key transcriptional targets by RT-qPCR (*ATF3, DDIT3*/CHOP, *DNAJB1*) that are induced by treatment with Pano and LTI6426 and propose that these biomarkers could serve as potential pharmacodynamic (PD) markers of response to this new therapeutic regimen in clinical development. This work has translational significance as it suggests that the incorporation of LTI6426 into Pano regimens could capture previously unrealized activity of Pano and allow dose reduction to alleviate dose-limiting toxicities for MM patients.

## Materials and methods

### Cell lines and reagents

Cell lines (KMS11, OPM2, and ANBL6) were purchased from American Type Culture Collection (Manassas, VA, USA). MM.1S BzR cells were a gift from Dr. Brian Van Ness (U. of Minnesota) and have been described previously [25]. LTI6426 was provided by Leukogene Therapeutics, Inc. (Charleston, SC) with purity and identity that was confirmed by liquid chromatograph–mass spectrometry (LC–MS) and nuclear magnetic resonance (NMR) as shown previously [16]. Bortezomib (Catalog No. S1013), carfilzomib (Catalog No. S2853), panobinostat (Catalog No. S1030), vorinostat (Catalog No. S1047), ricolinostat (Catalog No. S8001), entinostat (Catalog No. S1053), romidepsin (Catalog No. S3020), tazmetostat (Catalog No. S7128), ML324 (Catalog No. S7296), JQ1 (Catalog No. S7110), OTX015 (Catalog No. S7360), ABBV744 (Catalog No. S8723), IBET151 (Catalog No. S2780), and GSK-LSD1 (Catalog No. S7574) were purchased from Selleck Chemicals (Houston, TX, USA).

### Flow cytometry

Cells were treated as indicated, washed with ice-cold PBS, and collected. The cells were then fixed with the Fixation/Permeabilization Solution Kit from BD Biosciences (San, Jose, CA, USA) according to the manufacturer’s instructions. Cells were then treated with 0.125 mg/mL final concentration of rabbit anti-active caspase-3 (BD Pharmigen; San, Jose, CA, USA) and incubated at room temperature for 20 min. The cells were then washed with 1 mL of permeabilization/wash solution and resuspended in 50 µL of diluted Alexa Fluor 488 goat anti-rabbit IgG (Invitrogen; Carlsbad, CA, USA) according to the manufacturer’s instructions. The cells were incubated for 20 min at room temperature while protecting from light, washed with 1 mL of permeabilization/wash solution, and resuspended in 300 µL of permeabilization/wash solution and analyzed with the FITC channel of a NovoCyte flow cytometer (Acea Biosciences; San Diego, CA, USA).

### Determination of synergistic drug interactions

Cell viability was measured using CellTiter-Glo^®^ (Promega; Madison, WI, USA) and a Spectramax L microplate luminometer (Molecular Devices; San Jose, CA, USA). Synergy analysis was conducted as follows using principles of isobologram analysis: cell viability RLU values in the presence and absence of LTI6426 were independently determined and normalized to the zero Pano group to account for any effects of LTI6426 monotherapy. Thus, any separation of the curves indicates a true change in the sensitivity to Pano with a leftward shift in the curve indicating a synergistic drug interaction.

### Biomarker analysis by RT-qPCR

For quantitative reverse transcriptase polymerase chain reaction (RT-qPCR), total RNA was extracted using the RNeasy Plus Mini Kit (Qiagen; Hilden, Germany) and reversed transcribed and quantified using the Luna Universal One-Step RT-qPCR Kit (New England BioLabs; Ipswich, MA, USA). Primers: *ATF3* Fwd: 5ʹ-GGAGTGCCTGCAGAAAG-3ʹ, Rvs: 5ʹ-CCATTCTGA GCCCGGACAAT-3ʹ; *DNAJB1* Fwd: 5ʹ-CCAGTCACCCACGACCTTC-3ʹ, Rvs: 5ʹ-CCCTTCTTCACTTCGATGGTCA-3ʹ; *DDIT3* Fwd: 5ʹ-GGAAACAGAGTGGTCATTCCC-3ʹ, Rvs: 5ʹ-CTGCTTGAGCCGTTCATTCTC-3ʹ; *GAPDH* Fwd: 5ʹ-ACAACTTTGGTATCGTGGAAGG-3ʹ, Rvs: 5ʹ-GCCATCACGCCACAGTTTC-3ʹ; *FTH1* Fwd: 5ʹ-CGAGGTGGCCGAATCTTC C-3ʹ, Rvs: 5ʹ-GTTTGTGCAGTTCCAGTAGTGA-3ʹ; *FTL* Fwd: 5ʹ-CAGCCTGGTCAATTTGTACCT-3ʹ, Rvs: 5ʹ-GCCAATTCGCGGAAGAAGTG-3ʹ; *GCLC* Fwd: 5ʹ-GGCACAAGGACGTTCTCAAGT-3ʹ, Rvs: 5ʹ-CAGACAGGACCAACCGGAC-3ʹ; *GSTP1* Fwd: 5ʹ-CCCTACACCGTGGTCTATTTCC-3ʹ, Rvs: 5ʹ-CAGGAGGCTTTGAGTGAGC-3ʹ; *HERPUD1* Fwd: 5ʹ-CCGGTTACACACCCTATGGG-3ʹ, Rvs: 5ʹ-TGAGGAGCAGCATTCTGATTG-3ʹ; *HMOX1* Fwd: 5ʹ-AAGACTGCGTTCCTGCTCAAC-3ʹ, Rvs: 5ʹ-AAAGCCCTACAGCAACTGTCG-3ʹ; *HSPA6* Fwd: 5ʹ-GATGTGTCGGTTCTCTCCATTG-3ʹ, Rvs: 5ʹ-CTTCCATGAAGTGGTTCACGA-3ʹ; *NQO1* Fwd: 5ʹ-GAAGAGCACTGATCGTACTGGC-3ʹ, Rvs: 5ʹ-GGATACTGAAAGTTCGCAGGG-3ʹ; *NFE2L2* Fwd: 5ʹ-TCAGCGACGGAAAGAGTATGA-3ʹ, Rvs: 5ʹ-CCACTGGTTTCTGACTGGATGT-3ʹ; *TXN* Fwd: 5ʹ-GTGAAGCAGATCGAGAGCAAG-3ʹ, Rvs: 5ʹ-CGTGGCTGAGAAGTCAACTACTA-3ʹ; *TXNRD1* Fwd: 5ʹ-TAGGACAAGCCCTGCAAGACT-3ʹ, Rvs: 5ʹ-CCCCAATTCAAAGAGCCAATGT-3ʹ.

### In vivo studies

Animal experiments were conducted as described previously [17] under the approval of the Institutional Animal Care and Use Committee (IACUC) of the Medical University of South Carolina (MUSC, Protocol #2020-00915). NOD-SCID IL2Rγ^−/−^ (NSG) mice (Jackson Laboratory; Bar Harbor, ME, USA) were injected with 100 mg/kg cyclophosphamide i.p. (200 µL total in DPBS). Two days later mice were injected with 1 × 10^6^ myeloma cells (MM.1S or MM.1S BzR) (100 µL total in DPBS) in the lateral tail vein. Mice were randomly assigned to treatment groups (*N* = 10) and dosed with drugs as indicated and vehicle contained 0.5% (v/v) DMSO and 5% (v/v) Kolliphor EL (200 µL total in DPBS). Treatments were initiated on day 14 post injection of MM cells. Treatments were as follows: (1) vehicle, (2) oral LTI6426 (10 mg/kg/day, continuous) (3) the combination of Btz (0.4 mg/kg, days 1, 3, 5, and 8, i.p.) and Pano (4 mg/kg, days 1, 3, 5, 8, 10, and 12, i.p.), and (4) a triple treatment of LTI6426, Btz, and Pano in 21-day cycles. For experiments quantifying the number of MM plasma cells in the bone marrow of mice, hind leg femurs were harvested, stripped of soft tissue, and marrow exposed by a sagittal cut with a sharp blade. Bone marrow cells were collected in cold PBS, stained with a PE-conjugated anti-CD138 antibody (Miltenyi Biotec; Bergisch Gladbach, Germany), and quantified by flow cytometry. For statistical power calculations, a sample size of 10 mice per group yielded 81% power to detect significant differences in survival rate (10% vs. 75%) between control and treatment groups at 8 weeks with Type I error α of 0.0167(= 0.05/3) based on the log-rank test. The assumption of 10% survival in the control group and 75% in the treatment group(s) was made based on our experience with this model. The Kaplan–Meier curve and a log-rank (Mantel–Cox) test were used to analyze survival related outcomes.

## Results

### The indene PDI inhibitor, LTI6426, potentiates the anti-MM activity of Pano and other HDACi

**Fig. 1:**
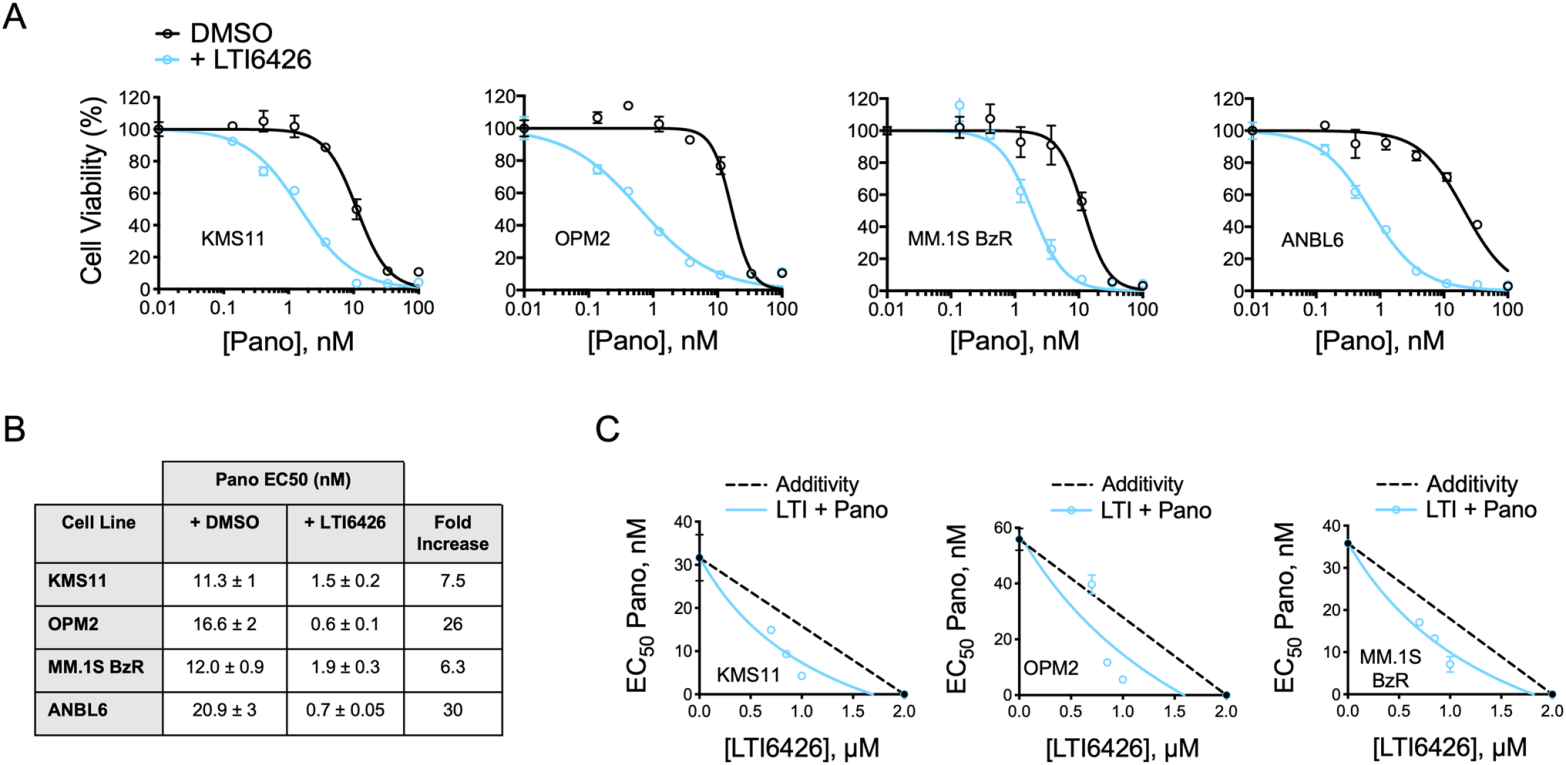
LTI6426 sensitizes MM cells to Pano. **A** The indicated MM cell lines were treated with a dose range of Pano for 48 h in the presence or absence of 1 µM LTI6426. The DMSO and LTI6426 data sets were independently normalized to the zero Pano treatment group to account for any cell death caused by LTI6426 treatment alone, which was less than 50% for the four indicated cell lines. Thus, any separation of the curves indicates a synergistic effect of LTI6426 on Pano sensitivity. **B** Quantitative data are shown for the dose response experiments conducted in **A**. The Effective Concentration 50 (EC_50_), or the concentration required to kill 50% of the cells, was extrapolated from each Pano dose response curve (*N* = 3) for cells treated with DMSO (control) and LTI6426. The Fold Increase indicates the degree to which LTI6426 enhanced Pano sensitivity and was determined by dividing the Pano EC_50_ in the control group by the Pano EC_50_ in the LTI6426 treatment group. **C** Isobologram analysis was conducted for the indicated cell lines. The dashed black line indicates the no effect/additive isobole. The leftward shift of the LTI6426 + Pano isobole indicates a superadditive/synergistic drug interaction

**Fig. 2:**
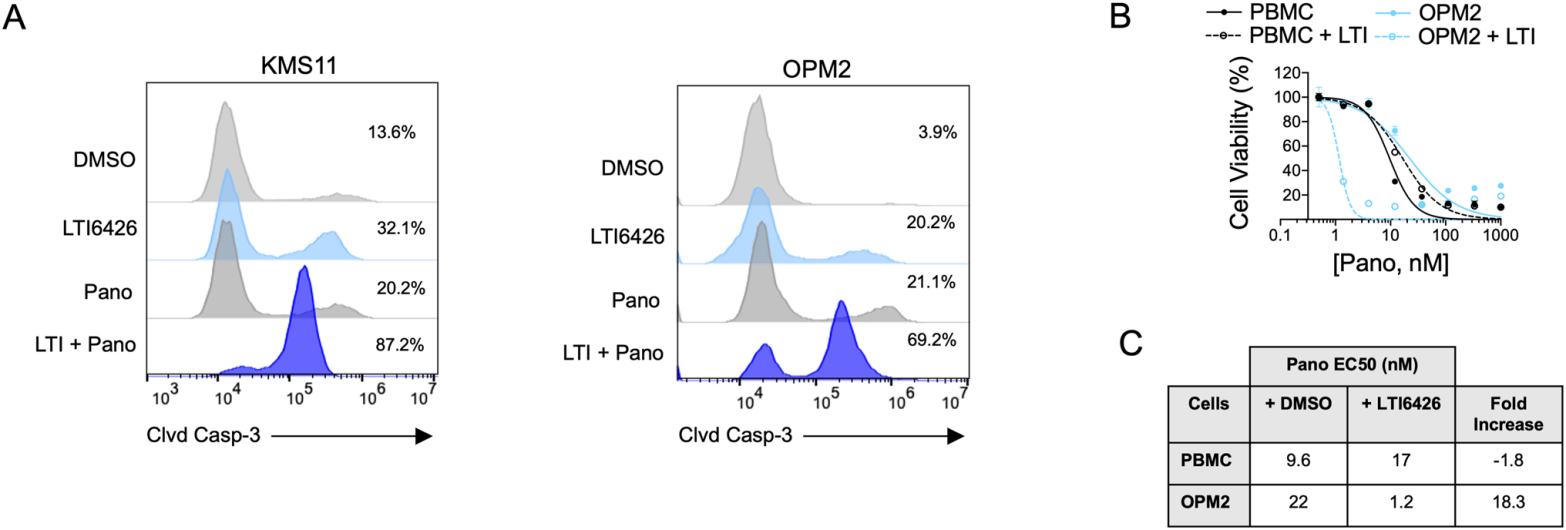
LTI6426 potentiates Pano-induced apoptosis in MM cells, and does not enhance Pano cytotoxicity in normal human PBMCs. **A** KMS11 and OPM2 cells were treated with DMSO (vehicle control), 1 µM LTI6426, 50 nM Pano, or the combination for 24 h. Cleaved caspase-3 (Clvd Casp-3) was analyzed as a marker of apoptotic cell death by flow cytometry. The percentage of cells that were positive for Clvd Casp-3 is shown. **B** Cell viability data are shown for normal human PBMCs that were treated with LTI6426 (1 µM) and a dose range of Pano for 48 h. Data were analyzed as described in (Fig. 1A) where overlapping Pano dose response curves indicate additive effects or no drug interactions, whereas a leftward shift indicates synergy and a rightward shift indicates antagonism. OPM2 MM cells were included for comparison and demonstrate a synergistic leftward shift in the dose response curve. The slight rightward shift in the curve for PBMCs indicates antagonism between LTI6426 and Pano. **C** Quantitative data are shown for the dose response experiments conducted in **B** using normal human PBMCs and OPM2 MM cells. The EC_50_ was extrapolated from each Pano dose response curve (*N* = 3) for cells treated with DMSO (control) and LTI6426. The Fold Increase indicates the degree to which LTI6426 enhanced Pano sensitivity and was determined by dividing the Pano EC_50_ in the control group by the Pano EC_50_ in the LTI6426 treatment group. Note that the fold change for PBMCs is negative, indicating that LTI6426 not only failed to increase the sensitivity of PMBCs to Pano, but modestly reduced sensitivity

We previously reported the discovery, medicinal chemistry optimization, and the potency and selectivity of an alkenyl indene class of PDI inhibitor (LTI6426; previously E64FC26) in biochemical assays and MM and solid tumor models [16–18]. To explore the potential of an LTI6426 and Pano combination in MM, we screened a panel of MM cell lines for synergistic cytotoxic activity. Cell models included KMS-11 and OPM2, which represent the high risk t(4;14) cytogenetic subclass [26, 27], proteasome inhibitor resistant MM.1S BzR cells, and ANBL6 cells, which others have shown are a close genomic representation of primary patient MM [28]. We observed true synergy between LTI6426 and Pano in all four cell lines, as indicated by the leftward shift of the Pano cell viability dose curve in the presence of LTI6426. The Effective Concentration 50 (EC_50_) values for Pano decreased in the presence of LTI6426 from 11.3 to 1.5 nM (a 7.5-fold increase in sensitivity), 16.6–0.6 nM (26-fold), 12.0–1.9 nM (6.3-fold), and 20.9–0.7 nM (30-fold) in KMS-11, OPM2, MM.1S BzR, and ANBL6 cells, respectively (Fig. 1A, B). Isobologram analysis was conducted and confirmed the synergistic nature of the drug interaction as indicated by the leftward shift of the LTI6426/Pano curve relative to the no effect/additive isobole (Fig. 1C). The synergy was largely due to apoptotic cell death as the combination synergistically increased caspase-3 cleavage/activation (Fig. 2A). In KMS11 cells, cleaved caspase-3 (CC3) levels increased by 73% in the combination group compared to the predicted additive effect of 27% that would have resulted if LTI6426 (20%) and Pano (7%) monotherapies were interacting in a purely additive fashion. Likewise, in OPM2 cells, the individual LTI6426 (17%) and Pano (17%) monotherapies would have generated a predicted 34% increase in CC3 if the effect were additive, but the actual increase was 66%, indicating a synergistic effect. This synergy was absent in normal human peripheral blood mononuclear cells (PBMCs), where LTI6426 had an antagonistic effect on Pano, reducing sensitivity by 1.7-fold (9.6 nM–17 nM, *p* = 0.0111, Fig. 2B, C). Taken together, PDI inhibition with LTI6426 enhances Pano-induced MM cell death and apoptosis in a heterogenous group of MM cell models. This combination is truly synergistic in MM cells but not normal PBMCs, suggesting this is not a generalized effect but one that is dependent on the biology of MM cells.

**Fig. 3:**
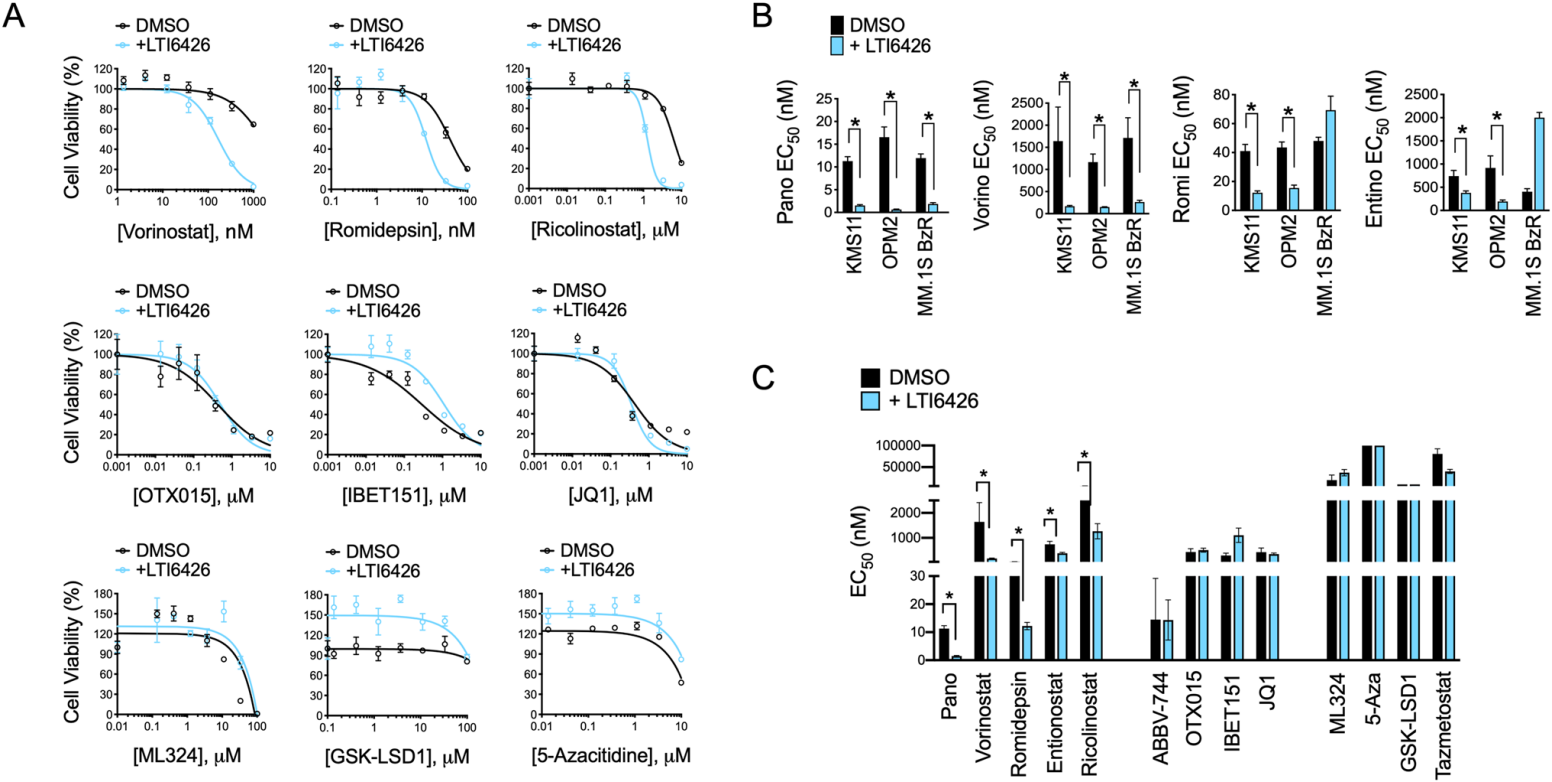
LTI6426 enhances the effects of HDACi but not other epigenetic modifying drugs. **A** KMS11 cells were treated with an 8-dose range of the indicated epigenetic drugs in the absence (DMSO) or the presence of 1 µM LTI6426. Dose response curves are shown. Overlapping curves indicate no effect or additive drug interactions whereas a leftward shift indicates synergy and rightward shift indicates antagonism. **B** LTI6426 sensitization of the HDACi Pano, vorinostat, romidepsin, and entinostat is shown for the indicated cell lines. EC_50_ values (in nM) for each of the indicated HDACi in the presence of DMSO (control, black bars) or 1 µM LTI6426 (blue bars) are provided. EC_50_ were calculated as described in (Fig. 1A) where any effects of LTI6426 are normalized to isolate its impact on HDACi sensitivity. **C** KMS11 cells were treated with a dose range of the indicated epigenetic drugs in the absence (black bars) or presence (blue bars) of 1 µM LTI6426. EC_50_ values from the individual dose response curves are shown. *p < 0.05 by student’s *t* test

We next tested whether the synergy between LTI6426 and Pano was observed with other epigenetic drugs in MM models. Indeed, a synergistic drug interaction was evident with other HDACi, including the pan-HDACi, vorinostat, and the isoform selective HDACi, ricolinostat (HDAC6) [29], romidepsin (class I HDACs) [30], and entinostat (class I HDACs) [31] (Fig. 3A, B). MM.1S BzR was the exception, showing synergy between LTI6426 and only the pan-HDACi, Pano and vorinostat. Negligible synergy (< twofold) was observed for combinations of LTI6426 and other classes of epigenetic modifying drugs (Fig. 3A and C). The drugs we tested included inhibitors of lysine specific demethylase 1 (LSD1, GSK-LSD1), EZH2 (tazmetostat), Jumonji domain containing demethylase 2 (JMJD2, ML324), the demethylating agent, 5-Azacitidine, and BET/bromodomain inhibitors (ABBV-744, OTX015, IBET151, and JQ1). These results indicate that LTI6426 enhances the anti-MM activity of HDACi, but not all epigenetic drugs. This further suggests that molecular events downstream of PDI and HDAC inhibition converge in a specific manner to drive MM cell death.

### ATF3, DDIT3/CHOP, and DNAJB1 induction characterize the molecular response to LTI6426/Pano combinations

**Fig. 4:**
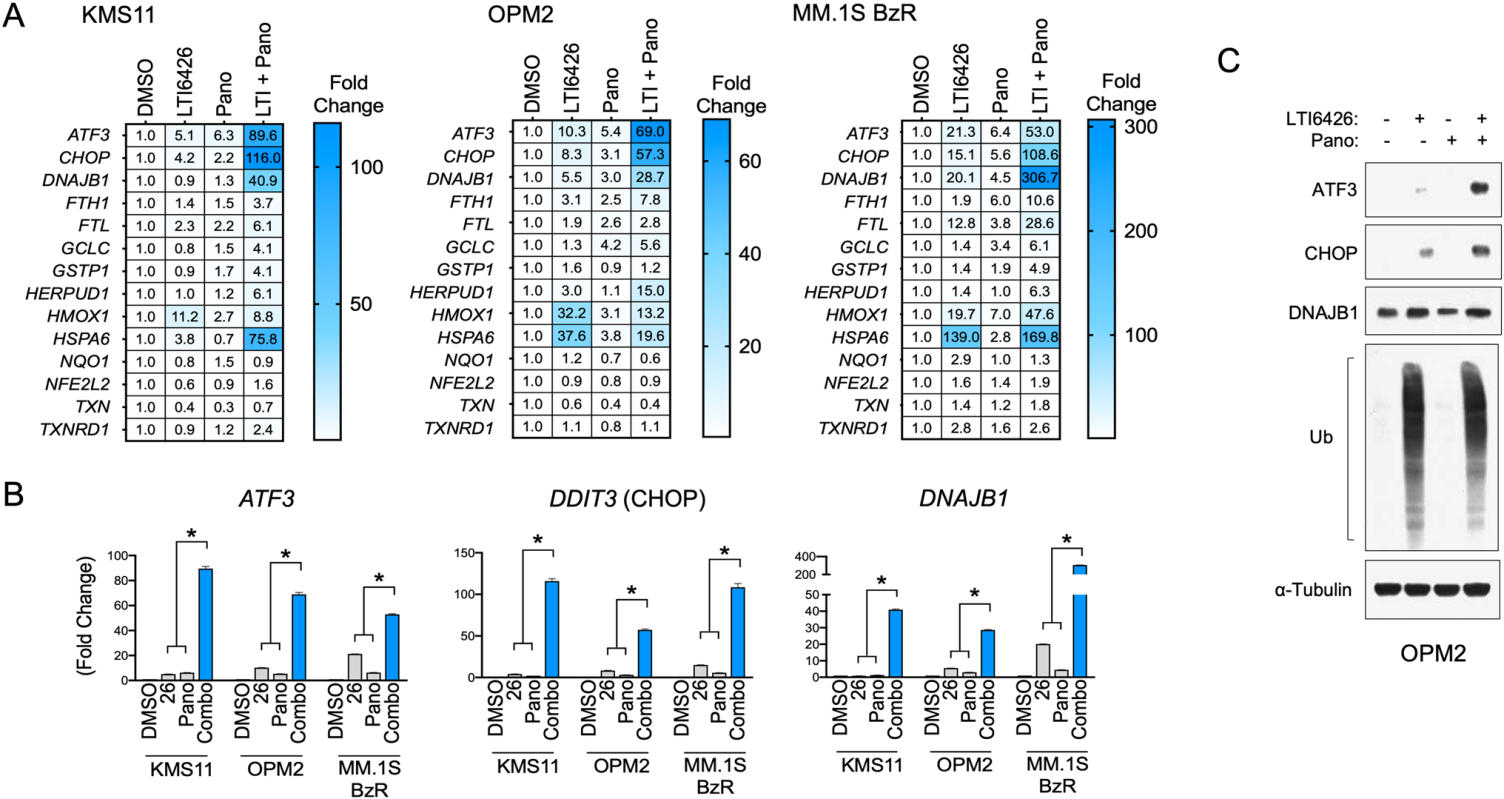
LTI6426 + Pano induces an ER stress transcriptional response characterized by synergistic *ATF3, DDIT3/*CHOP, and *DNAJB1* induction. **A** The indicated UPR/ER stress pathway gene targets were analyzed by RT-qPCR. KMS11, OPM2, and MM.1S BzR MM cells were treated with DMSO (vehicle control), 1 µM LTI6426, 50 nM Pano, or the combination for 20 h. RNA was extracted and RT-qPCR runs were performed in duplicate and fold change in expression levels were determined using glyceraldehyde 3-phosphate dehydrogenase (*GAPDH)* as an internal reference. Fold change data are shown relative to the DMSO control. **B** The indicated cell lines were treated as in **A** and the *ATF3, DDIT3/*CHOP, and *DNAJB1* mRNA transcript levels were analyzed by RT-qPCR. *p < 0.01 by student’s *t* test comparing LTI6426 + Pano (L + P) to LTI6426 and Pano independently (*N* = 3). **C** OPM2 cells were treated with 1 µM LTI6426, Pano (50 nM), or the combination for 24 h. Western blots are shown

We next set out to explore and characterize the molecular underpinnings of the synergy between LTI6426 and Pano in MM cells. Our previous work demonstrated a convergence of HDACi on the unfolded protein response (UPR)/ER stress and oxidative stress transcriptional programs that are activated in response to PDI inhibition [16–18]. To evaluate the effects of the combination on these two stress pathways, we constructed an array of gene targets from UPR/ER stress (*ATF3, DDIT3, HERPUD1, HSPA6,* and *DNAJB1)* and oxidative stress pathways (*FTH, FLT, HMOX1, NQO1, GCLC, GSTP1, NFE2L2, TXN, and TXNRD1*). We then measured the expression of these gene targets by RT-qPCR after treatment with LTI6426, Pano, or the combination. Single agent LTI6426 induced an ER stress and oxidative stress signature predominantly characterized by up-regulation of *ATF3*, *DDIT3*/CHOP, *HSPA6*, *DNAJB1*, and *HMOX1* in all three cell lines that were evaluated (Fig. 4A). Single agent Pano induced minimal effects on this gene set with *ATF3* being the only gene that was significantly induced by greater than fourfold in all three cell lines. Most importantly, the combination of LTI6426/Pano drove a synergistic induction of a small subset of these genes. Specifically, *ATF3, DNAJB1* and *DDIT3* (CHOP) gene induction was potentiated by the combination to a degree that far exceeded the sum of each individual treatment (Fig. 4A, B). For example, *ATF3* was induced by 89.6-fold in the LTI6426/Pano group compared to a predicted additive effect of 11.4-fold in KMS11 (*p* = 0.0004), 69-fold compared to 15.7-fold in OPM2 (*p* < 0.001), and 53-fold compared to a predicted 27.7-fold in MM.1S BzR cells (*p* < 0.001; Fig. 4B). Transcriptional effects were mirrored at the protein level, as the combination significantly increased ATF3 protein levels compared to the individual treatments (Fig. 4C). CHOP was also potentiated by the LTI6426/Pano combination at the protein level, although to a lesser extent than ATF3, whereas DNAJB1 protein was only modestly induced and there was no difference between LTI6426 monotherapy and the combination in OPM2 cells. As we have shown previously, LTI6426 induces a trademark smear of poly-ubiquitinated proteins due to protein folding errors caused by inhibition of PDI. Thus, the molecular effects of the LTI6426/Pano combination intersects at the transcriptional level to induce a unique gene expression signature characterized predominantly by *ATF3, DDIT3,* and *DNAJB1* induction. This suggests that these targets, particularly ATF3, may be key effectors in the synergistic anti-MM apoptotic response to the combination, and further identifies *ATF3, DDIT3/*CHOP, and *DNAJB1* mRNA are biomarkers of response to this new drug combination.

### LTI6426 enhances the anti-MM effects of Pano-based drug regimens in vivo

**Fig. 5:**
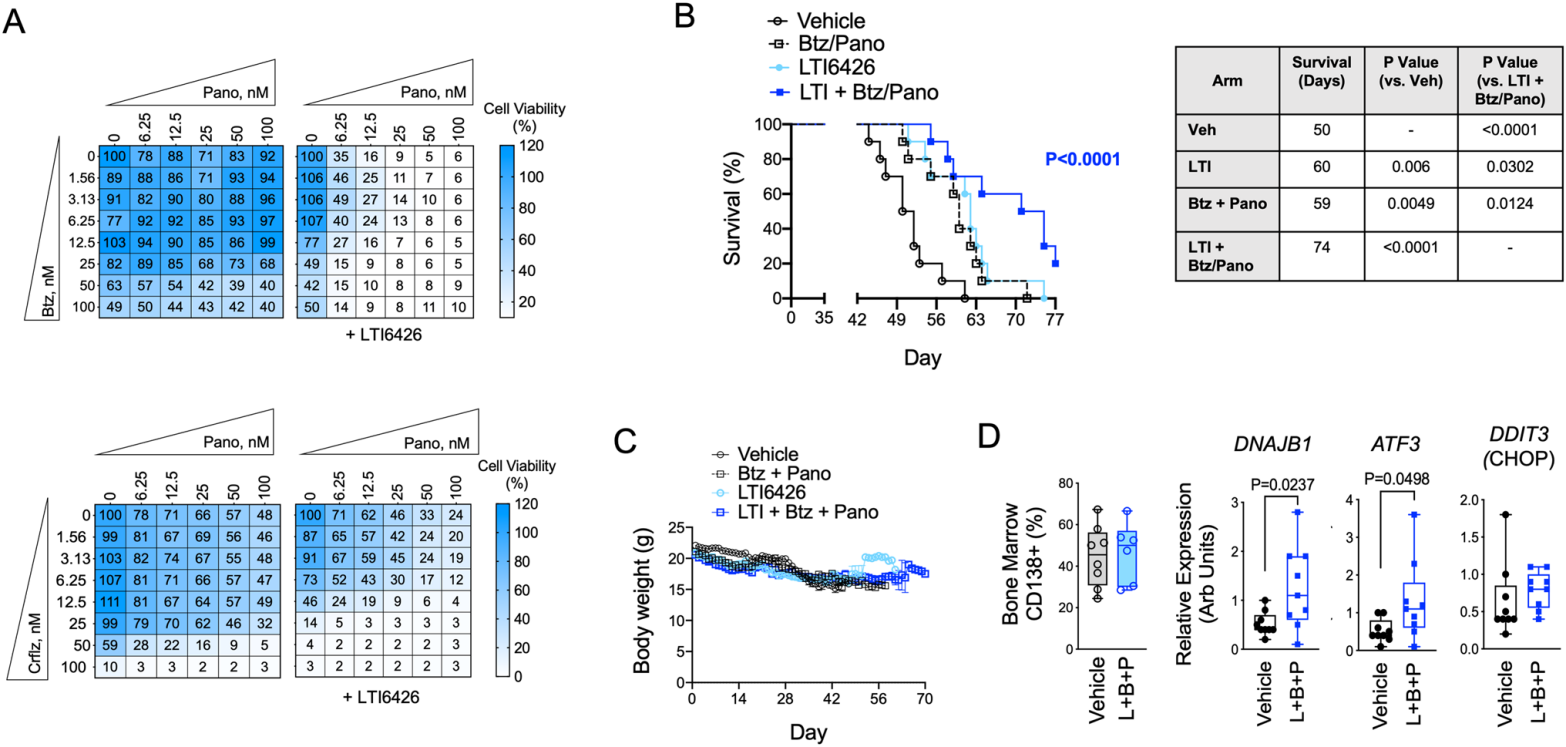
In vivo anti-MM efficacy of an LTI6426, Btz, and Pano triple treatment. **A** (Top) Dose matrices are shown for in vitro cytotoxicity analysis of Pano and bortezomib (Btz) in the absence and presence of 1 µM LTI6426. KMS-11 cells were treated for 24 h and cell viability was measured. Data from the no LTI6426 and + LTI6426 groups were analyzed independently to provide a measurement of the relative effects on Pano and Btz, alone and in combination. (Bottom) Identical experiments were conducted using the second generation proteasome inhibitor carfilzomib (Crflz) instead of Btz. **B** NOD-SCID-IL-2Rγ-/- (NSG) mice were injected systemically via the lateral tail vein with 1 × 10^6^ MM.1S BzR cells, a route of injection that we’ve shown previously promotes colonization of the mouse bone marrow by MM cells and a pathology that closely resembles human MM [17]. Mice were randomly assigned to the four indicated treatment groups and survival endpoints were determined by a blinded investigator. LTI6426 was dosed continuously, daily at 10 mg/kg (p.o.), Pano was dosed at 4 mg/kg (i.p.) on days 1, 3, 5, 8, 10, and 12 of a 21-day cycle, and Btz was dosed at 0.4 mg/kg (i.p.) on days 1, 3, 8, 11 of a 21-day cycle. Vehicle control received a placebo consisting of DMSO/Kolliphor EL/PBS. Survival data are shown on the left (*N* = 10 mice per group). The table (right) shows median survival data with p-values for statistical significance that were determined by the Log-rank (Mantel-Cox) test. **C** Body weight data for the mice treated in (Fig. 5B) is shown. There were no statistically significant differences in body weight between the different treatment groups throughout the duration of the experiment. **D** RT-qPCR analysis of *DNAJB1*, *ATF3*, and *DDIT3* were conducted in bone marrow from mice that were treated with vehicle or the LTI6426/Btz/Pano triple treatment. Bone marrow was harvested from femurs as mice reached the survival endpoint. (*Left*) At the time of death, which was a median day 50 for vehicle and 74 for triple treatment, the number of MM plasma cells in the bone marrow was not significantly different between the groups as determined by flow cytometry using a human CD138 antibody (44.7 ± 5.2 vs. 46.6 ± 5.9, *p* = 0.8135, *N* = 10). All mice received final vehicle or triple (L + B + P) treatments 48 h prior to the endpoint. (*Right)* RT-qPCR runs were performed for the indicated gene targets in duplicate and changes in expression levels were determined by using *GAPDH* as a reference. Statistical significance was determined using a student’s *t* test

We next evaluated the potential of LTI6426/Pano combination in a human xenotransplant preclinical model of MM. This model incorporates intravenous injection of proteasome inhibitor resistant MM.1S BzR cells and faithfully models the human pathology of relapsed/refractory MM with colonization of the bone marrow by CD138 + MM plasma cells and poor sensitivity to Btz therapy [17]. As Pano is indicated for relapsed/refractory MM in a regimen with Btz, we randomized mice into groups that would receive (1) vehicle, (2) oral LTI6426 (10 mg/kg/day, continuous) (3) the combination of Btz (0.4 mg/kg, days 1, 3, 5, and 8, i.p.) and Pano (4 mg/kg, days 1, 3, 5, 8, 10, and 12, i.p.), and (4) a triple treatment of LTI6426, Btz, and Pano in 21-day cycles. In vitro experiments were first conducted and showed a synergistic effect of LTI6426 on Btz and Pano, both independently and as a triple treatment (Fig. 5A). Similar experiments showed a comparable three-way synergy between LTI6426, Pano, and the second generation proteasome inhibitor, carfilzomib (Crflz, Fig. 5A). In vivo*,* the triple treatment of LTI6426, Btz, and Pano was highly efficacious, increasing median survival from 50 days (vehicle control group) to 74 days (*p* < 0.0001; *N* = 10; Fig. 5B). The individual treatment arms also increased median survival. The Btz/Pano treatment cohort showed an improvement in survival of 9 days compared to vehicle (*p* = 0.0049, *N* = 10) and the LTI6426 monotherapy improved median survival by 10 days (*p* = 0.006, *N* = 10). However, survival in the individual treatment cohorts was inferior when compared to the triple treatment of LTI6426/Btz/Pano (p = 0.0124 for Btz/Pano vs. LTI6426/Btz/Pano and *p* = 0.0302 for LTI6426 vs. LTI6426/Btz/Pano). All treatments were well tolerated as body weight remained constant throughout the experiment (Fig. 5C), and animals did not exhibit any overt visual or behavioral signs of distress. Notably, we observed a strong effect of the combination even though Pano was given at a dose of 4 mg/kg on days 1, 3, 5, 8, 10, and 12 of a 21-day cycle. The dose and dosing schedule we chose was lower and less frequently administered than what was used in published studies of Pano. Doses in these previous studies ranged from 5–25 mg/kg given on a continuous daily schedule [32–34]. Given that *DNAJB1, ATF3* and *DDIT3* were biomarkers of the LTI6426/Pano combination response in cell models, we next asked whether those markers were induced in vivo. Bone marrow was harvested from femurs of mice from vehicle and LTI6426 + Btz/Pano treatment groups as they reached the survival endpoint. Animals were dosed with the final vehicle or triple treatment 48 h prior to the survival endpoint. RNA was extracted from an equal number of MM plasma cells in vehicle and treatment groups (Fig. 5D, left panel) and human *DNAJB1, ATF3,* and *DDIT3* transcript levels were measured by RT-qPCR*.* Similar to what we observed in cell culture models, *DNJAB1* and *ATF3* expression were significantly higher in the LTI6426 + Btz/Pano group (*p* = 0.0237 and 0.0498, respectively, *N* = 9; Fig. 5D, right panels). *DDIT3/*CHOP levels showed an increasing trend, although the difference between the groups did not reach statistical significance. We conclude that LTI6426 enhances the activity of a clinically relevant Pano regimen in vivo, and this only required low Pano doses that were well tolerated in mice with the inclusion of other MM standard of care agents (i.e., Btz). The key transcriptional networks that were activated in response to this synergistic combination using cell model systems were found to be up-regulated in MM plasma cells from the bone marrow of mice treated with LTI6426 + Btz/Pano mice. These molecular findings provide a mechanistic understanding of the drug synergy and offer candidate biomarkers for assessing response and pharmacodynamics.

## Discussion

Epigenetic dysregulation is a hallmark of malignant cells. In response to this observation, therapeutic platforms (*e.g*., HDACi, BET/Bromodomain inhibitors, etc.) were developed to normalize the epigenome for clinical gain. Multiple HDACi (Pano, vorinostat, romidepsin, belinostat) have been approved for the treatment of cancer, although their impact has been limited to cutaneous and peripheral T-cell lymphoma (CTCL and PTCL) and MM [5, 35–37], which account for less than 1% of all cancers [38]. In MM, Pano is used sparingly in the clinic despite FDA approval and its high potency and specificity for HDACs. For example, a study in MM patients from Japan showed that only a small fraction of eligible patients were prescribed Pano compared to other novel agents [39]. A comparable study based on electronic health records between 2011 and 2019 showed a similar trend in the US [40]. This is due in part to the high grade AEs that are observed with the FDA-approved Pano/Btz/dexamethasone regimen [41]. However, it may also reflect the increasingly complex nature of the MM therapeutic paradigm and the fact that Pano is reserved for heavily pretreated late stage patients who have received, on average, four previous lines of therapy and are commonly refractory to a proteasome inhibitor and immunomodulatory agent (IMiD) [42]. In addition, the recent entry of several new therapeutic agents into the MM space further complicates if/how Pano might best be incorporated into regimens of those new agents. Recent studies have integrated Pano into regimens that eliminated dexamethasone (*i.e.,* steroid-sparing) and switch to the proteasome inhibitor carfilzomib [13, 14], which has a different toxicity profile than Btz. Early evidence suggests that this approach is efficacious and tolerable, perhaps allowing for increased flexibility in the Pano dosing and schedule. Our data show a strong synergy between LTI6426 and Pano at low doses of Pano, suggesting that this combination would permit Pano dose reduction and alleviate toxicity while maintaining the anti-MM activity. We used a low dose of Pano (4 mg/kg, i.p.) for 6 days of a 21-day cycle. It is difficult to accurately translate dose level between mice and humans; however, pharmacokinetic (PK) data from preclinical and clinical Pano studies suggest that drug exposure levels from our dosing regimen would have been far lower than what is achieved in humans. For example, a single i.p. dose of 10 mg/kg Pano, which is 2.5 times the dose we used, produced maximum blood plasma concentrations (*C*_max_) of approximately 860 pM (300 pg/ml) in C57BL/6 mice [43]. By comparison, plasma drug concentrations in humans were significantly higher in clinical PK studies, as a single oral dose of Pano at 20 mg generated *C*_max_ levels close to 60 nM (2100 pg/ml) [44, 45], or approximately 70-fold higher exposure in humans than mice. We can therefore conclude by inference that the combination of LTI6426, Btz, and Pano demonstrated a significant increase in animal survival using a Pano dose that produced systemic drug concentrations much lower than what is achievable in patients. Our analysis of LTI6426 metabolism revealed no overlap with Pano regarding hepatic cytochrome P450 (CYP) enzyme affinity, thereby ruling out drug-drug interactions as the source of synergy with Pano in vivo*.* CYP3A4 and to a lesser extent, CYP2D6, are the primary enzymes responsible for hepatic Pano metabolism [46]. LTI6426, by comparison, showed negligible affinity for both CYP3A4 and CYP2D6 in inhibition studies using liver microsomes. LTI6426 was a weak CYP3A4 inhibitor in vitro with an inhibitory concentration 50 (IC_50_) of > 100 μM (midazolam) and 18.2 μM (testosterone) and was a weak inhibitor of CYP2D6 with an IC_50_ of 10.5 μM (dextromethorphan). In depth safety and pharmacokinetic/pharmacodynamic studies evaluating LTI6426 + Pano combinations are required in large animals and ultimately humans to conclusively determine drug dosing and systemic exposure levels.

PDI regulates protein folding and redox homeostasis, two vulnerabilities of MM that are dictated by the biology of the plasma cell as a mass producer of Ig proteins [47, 48]. Indeed, we and others have shown that MM cells are highly sensitive to PDI inhibitors as single agents [17, 49]. The structure of IgG molecules includes 12 disulfide bonds and it has been shown that PDI associates with Ig molecules and is critical for their proper folding [50, 51]. As such, PDI inhibition leads to a dramatic spike in misfolded poly-ubiquitinated proteins and ER stress and oxidative stress biomarkers [16–18]. We set out to identify biomarkers of the LTI6426/Pano combination in MM cells, focusing on the ER and oxidative stress pathways. From a panel of ER and oxidative stress transcriptional targets we found that *ATF3*, *DDIT3* (CHOP), and *DNAJB1* were synergistically up-regulated in response to the combination. From this observation, we postulate that Pano alters chromatin topography by altering histone acetylation in way that promotes the transcription of *ATF3*, *DDIT3*, and *DNAJB1,* which are induced by PDI inhibition and subsequent ER stress pathway activation. In a previous study we conducted comprehensive molecular analysis that confirmed this mechanism in solid tumor types [18], and our current study demonstrates that this mechanism is broadly active across hematological as well as solid tumor types. The current study builds on the earlier work by confirming the induction of these markers by RT-qPCR in MM bone marrow aspirates from mice in our in vivo studies, suggesting that these gene targets may be useful pharmacodynamic (PD) markers in clinical applications. PD markers are increasingly being incorporated into early stage clinical trial design to confirm target engagement and proof-of-mechanism in patients, to optimize dosing, and to guide “go, no-go” development decisions [52, 53]. Therefore, *ATF3, DDIT3/*CHOP, and *DNAJB1* are candidate PD markers for future MM trials that layer LTI6426 onto Pano-based regimens. In conclusion, this study demonstrates the potential of PDI inhibitors like LTI6426 to enhance the anti-MM activity of Pano. The potentiating effects of LTI6426 on HDACi in MM cells may enhance Pano efficacy and allow for dose reductions that mitigate toxicities in heavily treated relapsed/refractory MM patient populations.

## Data Availability

All data generated or analyzed during this study are included in this published article.
